# Sodium-glucose cotransporter 2 inhibitors in heart failure with preserved ejection fraction: A meta-analysis of randomized controlled trials

**DOI:** 10.1016/j.ijcha.2022.101103

**Published:** 2022-08-11

**Authors:** Hidekatsu Fukuta, Hiromi Hagiwara, Takeshi Kamiya

**Affiliations:** a Core Laboratory, Nagoya City University Graduate School of Medical Sciences, Nagoya, Japan; b Department of Medical Innovation, Nagoya City University Graduate School of Medical Sciences, Nagoya, Japan

**Keywords:** Heart failure, Preserved ejection fraction, Sodium–glucose cotransporter 2 inhibitors, Meta-analysis

## Abstract

**Background:**

Nearly half of patients with heart failure (HF) have preserved ejection fraction (EF) and the mortality and morbidity of patients with HF with preserved EF (HFpEF) are high. Patients with HFpEF are often elderly and their primary chronic symptom is severe exercise intolerance that results in a reduced quality of life. Thus, improvement of exercise capacity and quality of life presents another important clinical outcome in HFpEF patients. Recent randomized controlled trials (RCTs) and meta-analyses of RCTs reported that sodium–glucose cotransporter 2 (SGLT-2) inhibitors improved cardiovascular (CV) outcomes in patients with HF with reduced EF. Although the effects of SGLT-2 inhibitors in HFpEF patients have been examined in multiple RCTs, results are inconsistent due partly to limited power. We aimed to conduct a meta-analysis of RCTs on the effects of SGLT-2 inhibitors in HFpEF patients.

**Methods and Results:**

The search of electronic databases identified 11 RCTs including 10,845 patients. In pooled analyses, SGLT-2 inhibitors reduced the risk of a composite of hospitalization for HF and CV death (hazard ratio [95 % CI] = 0.78 [0.70, 0.87], P_fix_ < 0.001). SGLT-2 inhibitors significantly increased 6-minute walk distance (weighted mean difference [95 % CI] = 18.0 [6.8, 29.3] m; P_fix_ = 0.002) and the Kansas City Cardiomyopathy Questionnaire Total Symptom Score (weighted mean difference [95 % CI] = 2.57 [0.19, 4.96] points; P_random_ = 0.035) and reduced plasma NT-pro B-type natriuretic peptide levels (weighted mean difference [95 % CI] = −60.16 [−82.99, −37.33] pg/ml; P_fix_ < 0.001) compared with control.

**Conclusion:**

The present meta-analysis suggests that SGLT-2 inhibitors may be beneficial for HFpEF patients, especially in diabetic patients.

## 1 Introduction

Nearly half of patients with heart failure (HF) in the community have preserved ejection fraction (EF) and the mortality and morbidity of patients with HF with preserved EF (HFpEF) are high [1], [2], [3], [4]. However, there is no established pharmacotherapy to improve survival in HFpEF patients [5], [6], [7], [8], [9], [10]. Patients with HFpEF are often elderly and their primary chronic symptom is severe exercise intolerance that results in a reduced quality of life. (QOL) [11], [12]. Thus, improvement of exercise capacity and QOL presents another important clinical outcome in HFpEF patients.

Recent randomized controlled trials (RCTs) and meta-analyses of the RCTs reported that sodium–glucose cotransporter 2 (SGLT-2) inhibitors improved cardiovascular (CV) outcomes in patients with HF with reduced EF (HFrEF) [13], [14], [15], [16]. Although the effects of SGLT-2 inhibitors in HFpEF patients have been examined in multiple RCTs [17], [18], [19], [20], [21], [22], [23], results are inconsistent due partly to limited power. Accordingly, we aimed to conduct a meta-analysis of RCTs examining the effects of SGLT-2 inhibitors in HFpEF patients.

## 2 Methods

This meta-analysis was performed and reported according to the preferred reporting items for systematic reviews and meta-analyses (PRISMA) [24]. The protocol for this meta-analysis was published elsewhere [25]. Studies on the effect of SGLT-2 inhibitors in HFpEF patients published until March 4, 2022 were identified using PubMed/Medline, Scopus, Cochrane Library, and Web of Science electronic databases. For search of the eligible studies, the following key words and Medical Subject Heading were used: *diastolic heart failure*, *heart failure with normal (preserved) ejection fraction*, *randomized, sodium–glucose cotransporter 2 inhibitor(s)*. Our literature search was limited to studies involving human subjects and those published in English. Additionally, we manually searched the references that were cited in other relevant publications. Studies were considered eligible if they; (1) included HFpEF patients; (2) were RCT; (3) used SGLT-2 inhibitors; (4) compared with usual medical therapy or placebo control group.

Primary outcomes of interest were CV outcomes including a composite of hospitalization for HF and CV death, hospitalization for HF, CV death, and all-cause death. Secondary outcomes of interest were the severity of HF. In the measures of the severity of HF, plasma B-type natriuretic peptide (BNP) levels, plasma *N*-terminal pro-BNP (NT-proBNP) levels, and exercise capacity assessed as 6 min-walk distance (6MWD) were extracted. Other outcomes of interest were QOL assessed as the Kansas City Cardiomyopathy Questionnaire Total Symptom Score (KCCQ-TSS) and hematocrit levels.

Information on the study and patient characteristics, methodological quality, intervention strategies, and clinical outcomes was systematically extracted separately by 2 reviewers (HF and HH). Disagreements were resolved by consensus. The Cochrane Risk of Bias tool was used to assess the quality of included RCTs [26].

For all analyses, Comprehensive Meta Analysis Software version 2 (Biostat, Englewood, NJ, USA) was used. For categorical outcomes, the pooled estimates of hazard ratio (HR) or odds ratio (OR) with 95 % CI were calculated. For continuous outcomes, the effect size for the intervention was calculated by the difference between the means of the intervention and control groups at the end of the intervention. When available, the mean difference with corresponding standard deviation (SD), standard error of the mean (SEM) or confidence interval (CI) was directly extracted from the article. When the outcome was reported as median (range and/or interquartile range), the mean and SD were estimated as previously reported [27]. If the outcome was measured on the same scale, the weighted mean difference (WMD) and 95 % CI were calculated. Otherwise, the standardized mean difference (SMD) and 95 % CI were calculated. For each outcome, heterogeneity was assessed using the Cochran’s Q and I^2^ statistic; for the Cochran’s Q and I^2^ statistic, a p value of < 0.1 and I^2^ > 50 %, were considered significant, respectively [28]. When there was significant heterogeneity, the data were pooled using a random-effects model, otherwise a fixed-effects model was used. The sensitivity analysis was performed separately for RCTs that included only diabetic patients, those that used EF ≥ 50 % for the diagnosis of HFpEF, those with longer (>1 year) follow-up, and those that used placebo as control.

## 3 Results

**Fig. 1:**
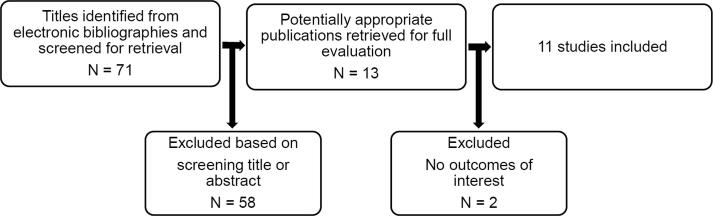
Selection process for studies included in meta-analysis. HFpEF indicates heart failure with preserved ejection fraction.

The study identification and selection process is summarized in Fig. 1. A total of 11 trials including 10845 patients were included in the present meta-analysis. The risk of bias summary is presented in supplemental Fig. 1.

The characteristics of the included trials and patients are presented in Table 1 and supplemental Table 1. Of the 11 included trials, 6 trials were HFpEF specific trials and 5 trials were those that included HFpEF patients. Eight trials included only diabetic patients and 3 trials included diabetic and non-diabetic patients. Definition of preserved EF ranged across trials from > 40 % to ≥ 50 %.

**Table 1:** Study characteristics.

Study, year	Region	Entry N, intervention/control	HFpEF specific trial	Definition of preserved EF	Diabetic status	Drug	Control	Follow-up	Outcomes of interest
DECLARE-TIMI 58 2019 [40]	Global	399/409	No	≥45 %	Diabetics	Dapagliflozin	Placebo	4.2 years	A, B, C, D
VERTIS CV 2020[41]	Global	680/327	No	>45 %	Diabetics	Ertugliflozin	Placebo	3.5 years	A, B, C, D
SCORED 2020 [42]	Global	843/824	No	≥50 %	Diabetics	Sotagliflozin	Placebo	16 months	A
SOLOIST WHF 2020 [43]	Global	127/129	No	≥50 %	Diabetics	Sotagliflozin	Placebo	9.2 months	A
CANDLE 2020 [44]	Japan	78/87	No	≥50 %	Diabetics	Canagliflozin	Glimepiride	24 weeks	G, I
MUSCAT-HF 2020 [17]	Japan	83/82	Yes	≥45 %	Diabetics	Luseogliflozin	Voglibose	12 weeks	F
CANONICAL 2021 [18]	Japan	42/40	Yes	≥50 %	Diabetics	Canagliflozin	Standard diabetic therapy	24 weeks	B
EMPEROR-Preserved 2021 [19]	Global	2997/2991	Yes	>40 %	Diabetics and non-diabetics	Empagliflozin	Placebo	26 months	A, B, C, D, G, H, I
PRESERVED-HF 2021 [21]	USA	162/162	Yes	≥45 %	Diabetics and non-diabetics	Dapagliflozin	Placebo	12 weeks	D, E, F, G, H
EMPERIAL-Preserved 2021 [22]	Global	157/158	Yes	>40 %	Diabetics and non-diabetics	Empagliflozin	Placebo	12 weeks	D, E, G, H
EXCEED 2022[23]	Japan	36/32	Yes	≥50 %	Diabetics	Ipragliflozin	Standard diabetic therapy	12 weeks	G

EF indicates ejection fraction; HFpEF, heart failure preserved ejection fraction.

A, a composite of hospitalization for heart failure (HF) and cardiovascular (CV) death; B, hospitalization for HF; C, CV death; D, all-cause death; E, 6-minute walk distance; F, plasma B-type natriuretic peptide levels; G, plasma *N*-terminal pro-B-type natriuretic peptide levels; H, the Kansas City Cardiomyopathy Questionnaire Total Symptom Score; I, hematocrit levels.

**Fig. 2:**
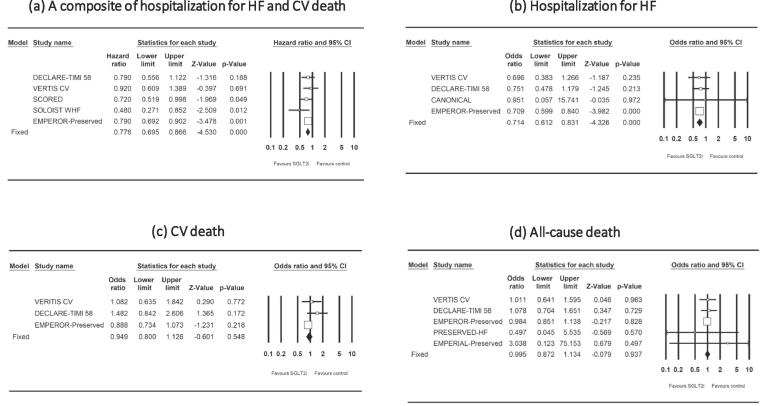
Forest plots showing the effects of sodium–glucose cotransporter 2 inhibitors (SGLT2i) on a composite of hospitalization for heart failure (HF) and cardiovascular (CV) death (a), hospitalization for HF (b), CV death (c), and all-cause death (d).

The effects of SGLT-2 inhibitors on CV outcomes in HFpEF patients are shown in Fig. 2. SGLT-2 inhibitors reduced the risk of a composite of hospitalization for HF and CV death (HR [95 % CI] = 0.78 [0.70, 0.87], P_fix_ < 0.001; heterogeneity, P = 0.46, I^2^ = 0 %) and the risk of hospitalization for HF (OR [95 % CI] = 0.71 [0.61, 0.83], P_fix_ < 0.001; heterogeneity, P = 0.99, I^2^ = 0 %). SGLT-2 inhibitors did not reduce the risk of CV death (OR [95 % CI] = 0.95 [0.80, 1.13], P_fix_ = 0.55; heterogeneity, P = 0.21, I^2^ = 36 %) or the risk of all-cause death (1.00 [0.87, 1.13], P_fix_ = 0.94; heterogeneity, P = 0.92, I^2^ = 0 %).

**Fig. 3:**
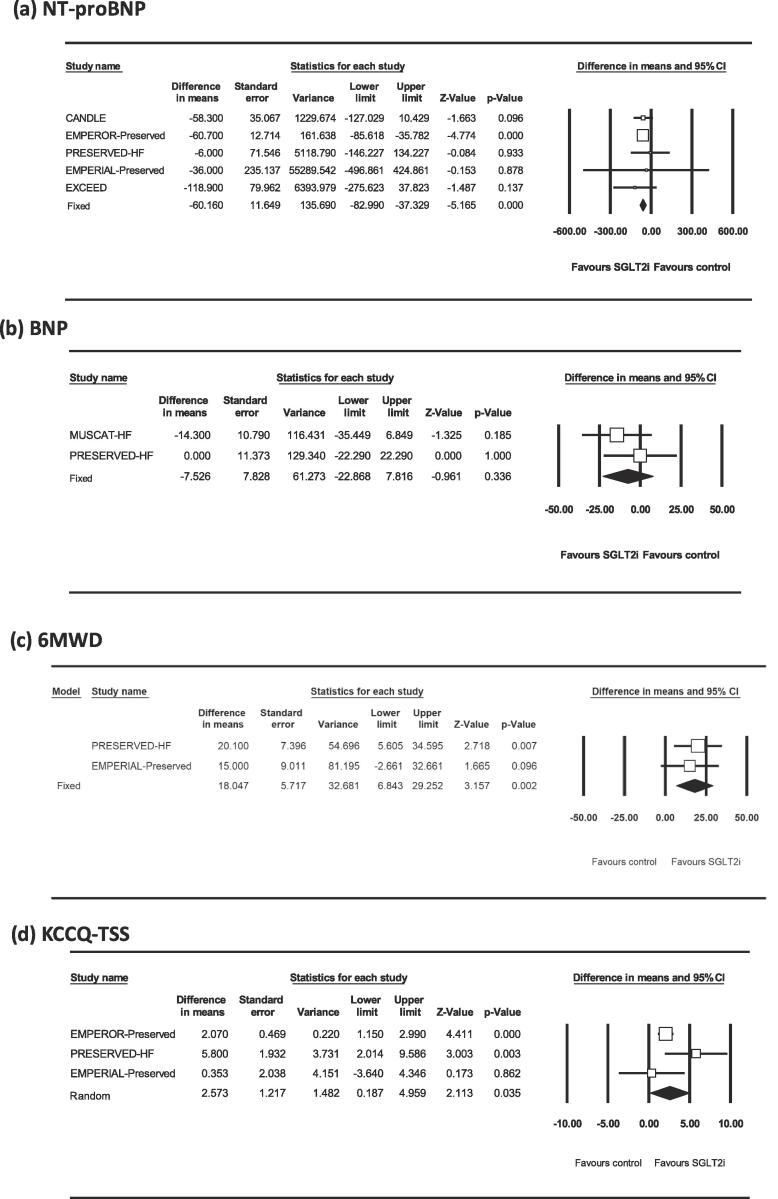
Forest plots showing the effects of sodium–glucose cotransporter 2 inhibitors (SGLT2i) on *N*-terminal pro-B-type natriuretic peptide levels (NT-proBNP; pg/ml; a), plasma B-type natriuretic peptide levels (BNP; pg/ml; b), 6-minute walk distance (6MWD; m; c), and the Kansas City Cardiomyopathy Questionnaire Total Symptom Score (KCCQ-TSS; points; d).

The effects of SGLT-2 inhibitors on NT-proBNP, BNP, 6MWD, and KCCQ-TSS in HFpEF patients are shown in Fig. 3. SGLT-2 inhibitors decreased NT-proBNP levels (WMD [95 % CI] = -60.16 [-82.99, −37.33] pg/ml; P_fix_ < 0.001; heterogeneity, P = 0.89, I^2^ = 0 %) and increased 6MWD (WMD [95 % CI] = 18.0 [6.8, 29.3] m; P_fix_ = 0.002; heterogeneity, P = 0.66, I^2^ = 0 %) and KCCQ-TSS (WMD [95 % CI] = 2.57 [0.19, 4.96] points; P_random_ = 0.035; heterogeneity, P = 0.11, I^2^ = 54 %) compared with control. SGLT-2 inhibitors did not change BNP levels compared with control (WMD [95 % CI] = −7.53 [–22.87, 7.82] pg/ml; P_fix_ = 0.34; heterogeneity, P = 0.36, I^2^ = 0 %).

The effect of SGLT-2 inhibitors on hematocrit is shown in supplemental Fig. 2. SGLT-2 inhibitors increased hematocrit levels compared with control (WMD [95 % CI] = 2.34 [2.16, 2.51] %; P_fix_ < 0.001; heterogeneity, P = 0.25, I^2^ = 23.6 %).

The results of sensitivity analysis are shown in supplemental Fig. 3-6. When pooled analysis was performed for RCTs that included only diabetic patients, SGLT-2 inhibitors reduced the risk of a composite of hospitalization for HF and CV death (HR [95 % CI] = 0.75 [0.62, 0.91], P_fix_ = 0.003; heterogeneity, P = 0.33, I^2^ = 12 %) but not the risk of all-cause death (OR [95 % CI] = 1.05 [0.77, 1.43], P_fix_ = 0.78 heterogeneity, P = 0.84, I^2^ = 0 %; supplemental Fig. 3). When pooled analysis was performed for RCTs that used EF ≥ 50 % for the diagnosis of HFpEF, SGLT-2 inhibitors reduced the risk of a composite of hospitalization for HF and CV death (HR [95 % CI] = 0.70 [0.56, 0.88], P_fix_ = 0.002; heterogeneity, P = 0.34, I^2^ = 6.8 %) but not the risk of all-cause death (OR [95 % CI] = 1.08 [0.70, 1.65], P_fix_ = 0.73; heterogeneity, P = 1.0, I^2^ = 0 %; supplemental Fig. 4). When pooled analysis was performed for RCTs with longer (>1 year) follow-up, SGLT-2 inhibitors reduced the risk of a composite of hospitalization for HF and CV death (HR [95 % CI] = 0.79 [0.71, 0.88], P_fix_ < 0.001; heterogeneity, P = 0.84, I^2^ = 0 %) but not the risk of all-cause death (OR [95 % CI] = 1.00 [0.87, 1.14], P_fix_ = 0.94; heterogeneity, P = 0.92, I^2^ = 0 %; supplemental Fig. 5). When pooled analysis was performed for RCTs that used placebo as control, SGLT-2 inhibitors decreased NT-proBNP levels (WMD [95 % CI] = −58.96 [-83.46, −34.46] pg/ml; P_fix_ < 0.001; heterogeneity, P = 0.75, I^2^ = 0 %) but not BNP levels compared with control (0.0 [–22.29, 22.29] pg/ml; P_fix_ = 1.0; heterogeneity, P = 1.0, I^2^ = 0 %; supplemental Fig. 6).

## 4 Discussion

In the present study, we conducted a meta-analysis of RCTs examining the effects of SGLT-2 inhibitors in HFpEF patients. We observed that 1) SGLT-2 inhibitors reduced the risk of hospitalization for HF; 2) SGLT-2 inhibitors reduced NT-proBNP levels; and 3) SGLT-2 inhibitors improved exercise capacity and QOL. These results suggest that SGLT-2 inhibitors may be beneficial for HFpEF patients.

Consistent with our meta-analysis, recent meta-analyses reported that SGLT-2 inhibitors reduced the risk of hospitalization for HF and the composite of hospitalization for HF and CV death but not the risk of CV death or all-cause death in HFpEF patients [29], [30], [31], [32], [33]. However, after these meta-analyses were conducted, the results of several important RCTs have been published [21], [22], [23]. Our meta-analysis confirms the reported effects of SGLT-2 inhibitors with a larger number of RCTs. To the best of our knowledge, our meta-analysis is the first to show the beneficial effects of SGLT-2 inhibitors on the severity of HF and QOL in HFpEF patients.

Inconsistent with the present study, the meta-analysis by Zhou et al reported that SGLT-2 inhibitors did not improve 6MWD in HFpEF patients [33]. The inconsistent results regarding the effect of SGLT-2 inhibitors on 6MWD appear to be due to different selection of RCTs for the pooled analysis. In our meta-analysis, 2 RCTs (the PRESERVED-HF [21] and the EMPERIAL-Preserved [22]) were included. However, the meta-analysis by Zhou et al included the EMPERIAL-Preserved but not the PRESERVED-HF. Instead, the meta-analysis by Zhou et al included the study by Ovchinnikov et al [34] which is written in Russian and thus was not included in our meta-analysis.

Although the present meta-analysis does not provide the mechanisms for the observed beneficial effects of SGLT-2 inhibitors in HFpEF patients, there is one possible explanation. The EMBRACE-HF study reported that SGLT-2 inhibitors decreased pulmonary artery diastolic pressure, a surrogate of left atrial pressure, in HF patients [35]. Several lines of evidence suggest that an increase in left atrial pressure may be the most important hemodynamic determinant of exercise capacity in HFpEF patients [36]. Thus, SGLT-2 inhibitors may ameliorate pulmonary congestion and can translate to improvements in the severity of HF and the risk of hospitalization for HF in these patients.

Atrial fibrillation is common in HFpEF patients and patients with HFpEF and AF have worse outcomes [37]. Although there are no RCTs examining the effect of SGLT-2 inhibitors for patients with HFpEF and AF, subgroup analyses of the EMPEROR-Preserved [19] and the EMPERIAL-Preserved [22] showed that SGLT2-inhibitos improved a composite of hospitalization for HF and CV death and exercise capacity similarly for HFpEF patients with AF and those without AF. Furthermore, subgroup analysis of the EMBRACE-HF reported that SGLT-2 inhibitors reduced pulmonary artery diastolic pressure similarly for HFpEF patients with AF and those without AF [35]. To confirm the reported potential benefit of SGLT-2 inhibitors for patients with HFpEF and AF, further trials specifically designed for patients with HFpEF and AF are necessary.

In the recent guidelines, addition of SGLT-2 inhibitors to optimal medical therapy is recommended to reduce the risk of CV death and worsening HF in patients with HFrEF, unless SGLT-2 inhibitors are contraindicated or not tolerated [38], [39]. However, there are no specific recommendations regarding the use of SGLT-2 inhibitors in HFpEF patients. Although recent meta-analyses including ours suggest that SGLT-2 inhibitors may be beneficial in HFpEF patients [29], [30], [31], [32], [33], further large trials are necessary to confirm the observed potential benefits of SGLT-2 inhibitors in these patients.

There are several limitations to the present study. First, our meta-analysis included the trials including only diabetic patients and those including both diabetic and non-diabetic patients (Table 1). The effects of SGLT-2 inhibitors were not analyzed separately for diabetic and non-diabetic patients. However, when the pooled analysis was performed for the RCTs that included only diabetic patients, the results substantially remained unchanged (supplemental Fig. 3). Second, our meta-analysis used subgroup results of RCTs including HF and non-HF patients [40], [41], [42], [43], [44] and thus might have introduced bias. Third, the number of the trials included in our meta-analysis was limited. Some of the outcomes of interest were not consistently reported, which might have introduced bias. There are several ongoing large trials examining effects of SGLT-2 inhibitors in HFpEF patients [45]. These trials will address the efficacy as well as safety of SGLT-2 inhibitors in these patients. Fourth, several of the trials included in our meta-analysis defined preserved EF as greater than or equal to 40 % or 45 % (Table 1), which is not consistent with a definition of HFpEF in the guidelines [46], [47]. However, even when the pooled analysis was performed for the RCTs that used EF ≥ 50 % for the definition of HFpEF, the results substantially remained unchanged (supplemental Fig. 4). In the subgroup analysis of EMPEROR-Preserved, the magnitude of the effect of empagliflozin on HF outcomes was similar in patients with EF > 45 % to < 65 % but was attenuated in patients with EF ≥ 65 % [48]. Further studies are necessary to examine whether SGLT-2 inhibitors differently affect HFpEF patients with different EF ranges. Finally, RCTs generally have strict enrollment criteria and patients with HFpEF are often elderly with many comorbidities [49]. Thus, the patients who participated in the RCTs in our meta-analysis might represent a selected group of patients that was poorly representative of patients treated in routine clinical practice. Further studies are necessary to examine whether our observed potential benefits of SGLT-2 inhibitors could be applied to real-world patients.

## 5 Conclusion

Our meta-analysis suggests that SGLT-2 inhibitors may reduce the risk of hospitalization for HF and may improve the severity of HF and QOL in HFpEF patients. Given the limited number of HFpEF specific trials in our meta-analysis, further large trials specifically designed for HFpEF are necessary to confirm our observed potential benefits of SGLT-2 inhibitors in HFpEF patients.

## Grant supporting this paper

This paper is not funded by any external source.

## Systematic review registration

INPLASY2021120033.

## Ethical approval

This article does not contain any studies with human participants performed by any of the authors.

## Declaration of Competing Interest

The authors declare that they have no known competing financial interests or personal relationships that could have appeared to influence the work reported in this paper.

## Appendix A Supplementary material

Supplementary Fig. 1 Risk of bias summary.

Supplementary Fig. 2 Forest plots showing the effect of sodium–glucose cotransporter 2 inhibitors (SGLT2i) on hematocrit levels (%; a).

Supplementary Fig. 3 Forest plots showing the effects of sodium–glucose cotransporter 2 inhibitors (SGLT2i) on a composite of hospitalization for heart failure (HF) and cardiovascular (CV) death (a) and all-cause death (b) for trials that included only diabetic patients.

Supplementary Fig. 4 Forest plots showing the effects of sodium–glucose cotransporter 2 inhibitors (SGLT2i) on a composite of hospitalization for heart failure (HF) and cardiovascular (CV) death (a) and all-cause death (b) for trials that used ejection fraction (EF) ≥50% for the diagnosis of heart failure with preserved EF.

Supplementary Fig. 5 Forest plots showing the effects of sodium–glucose cotransporter 2 inhibitors (SGLT2i) on a composite of hospitalization for heart failure (HF) and cardiovascular (CV) death (a) and all-cause death (b) for trials with longer (>1 year) follow-up.

Supplementary Fig. 6 Forest plots showing the effects of sodium–glucose cotransporter 2 inhibitors (SGLT2i) on N-terminal pro-B-type natriuretic peptide levels (NT-proBNP; pg/ml; a), plasma B-type natriuretic peptide levels (BNP; pg/ml; efb) for trials that used placebo as control.
