# Supplementary figures and images for: Sodium-glucose cotransporter 2 inhibitors in heart failure with preserved ejection fraction: A meta-analysis of randomized controlled trials
Source: Int J Cardiol Heart Vasc. 2022 Aug 11;42:101103. doi: 10.1016/j.ijcha.2022.101103 (PMC9399288; doi:10.1016/j.ijcha.2022.101103)

## Slide 1
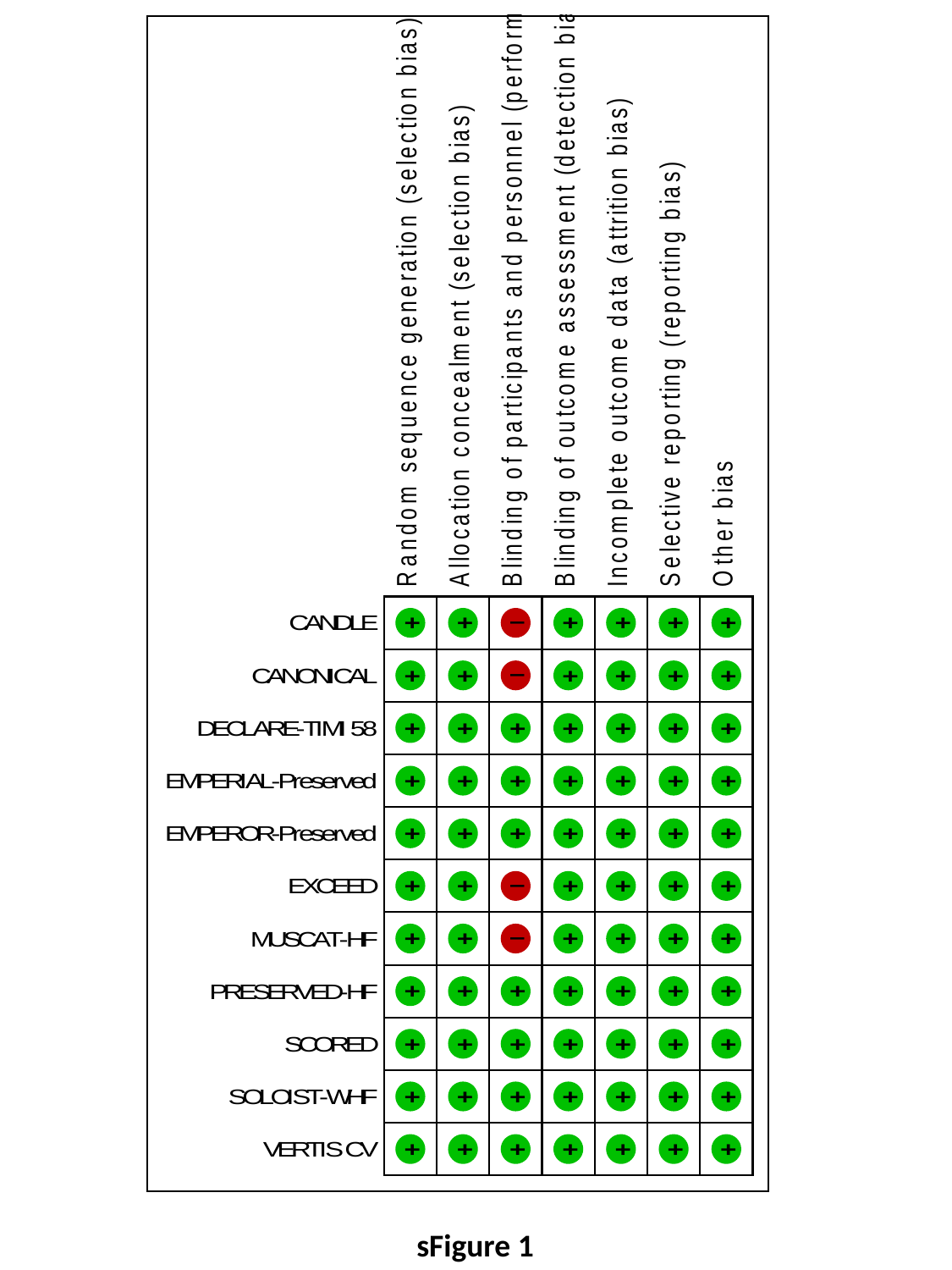

sFigure 1

Supplement: Supplementary Fig. 1 — Risk of bias summary. [file mmc1.pptx]

## Slide 1
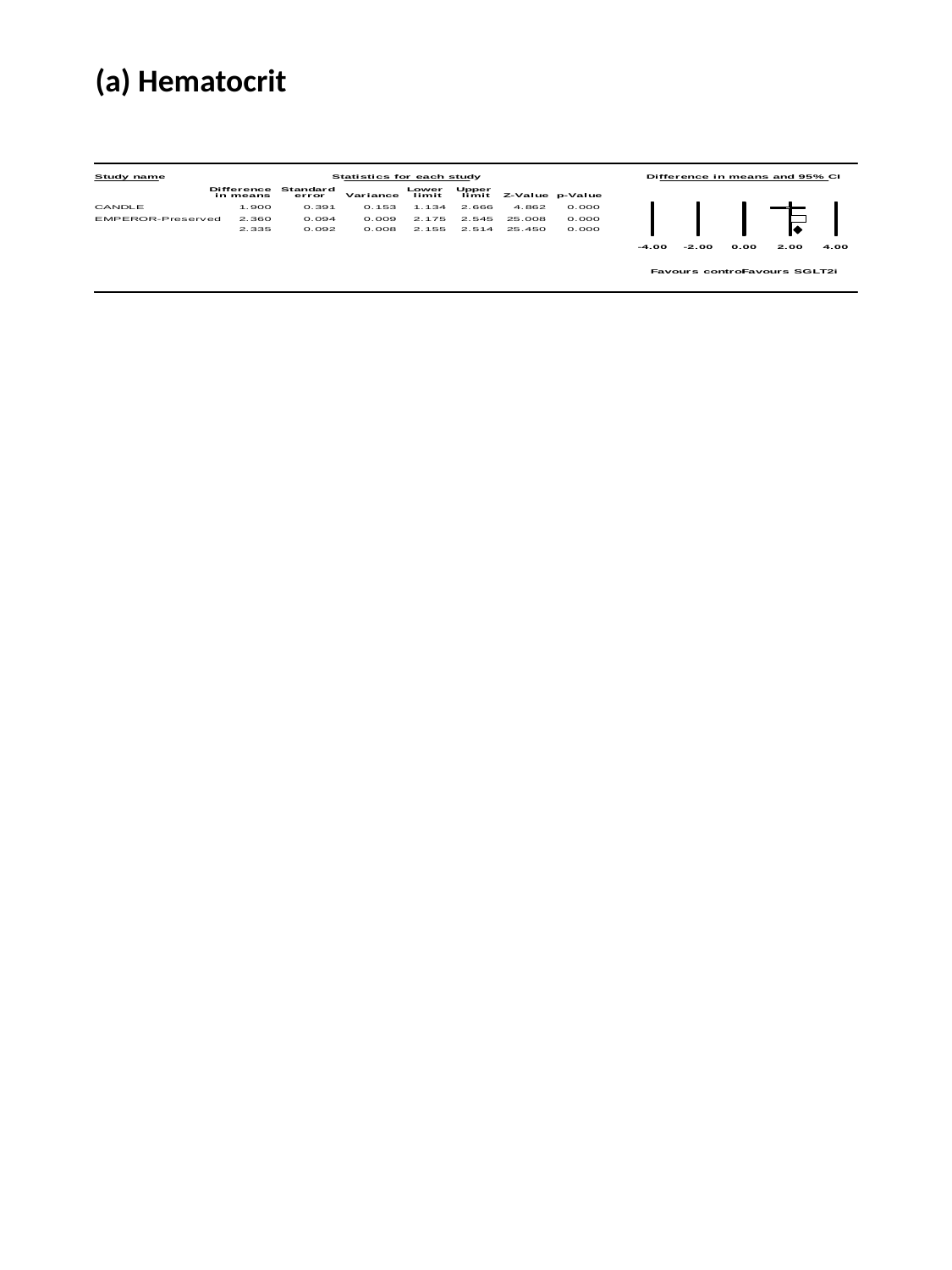

(a) Hematocrit

Supplement: Supplementary Fig. 2 — Forest plots showing the effect of sodium–glucose cotransporter 2 inhibitors (SGLT2i) on hematocrit levels (%; a). [file mmc2.pptx]

## Slide 1
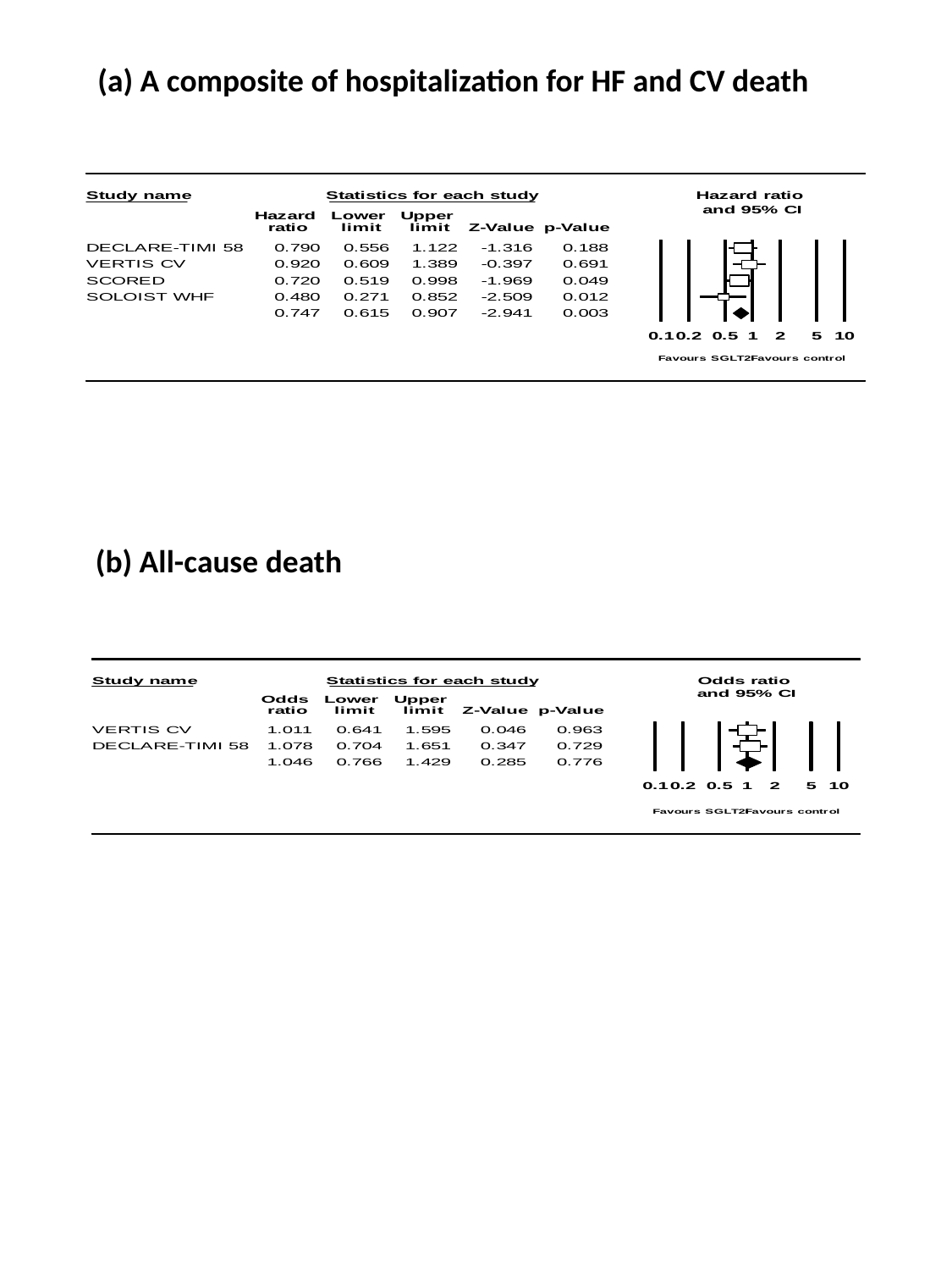

(a) A composite of hospitalization for HF and CV death
(b) All-cause death

Supplement: Supplementary Fig. 3 — Forest plots showing the effects of sodium–glucose cotransporter 2 inhibitors (SGLT2i) on a composite of hospitalization for heart failure (HF) and cardiovascular (CV) death (a) and all-cause death (b) for trials that included only diabetic patients. [file mmc3.pptx]

## Slide 1
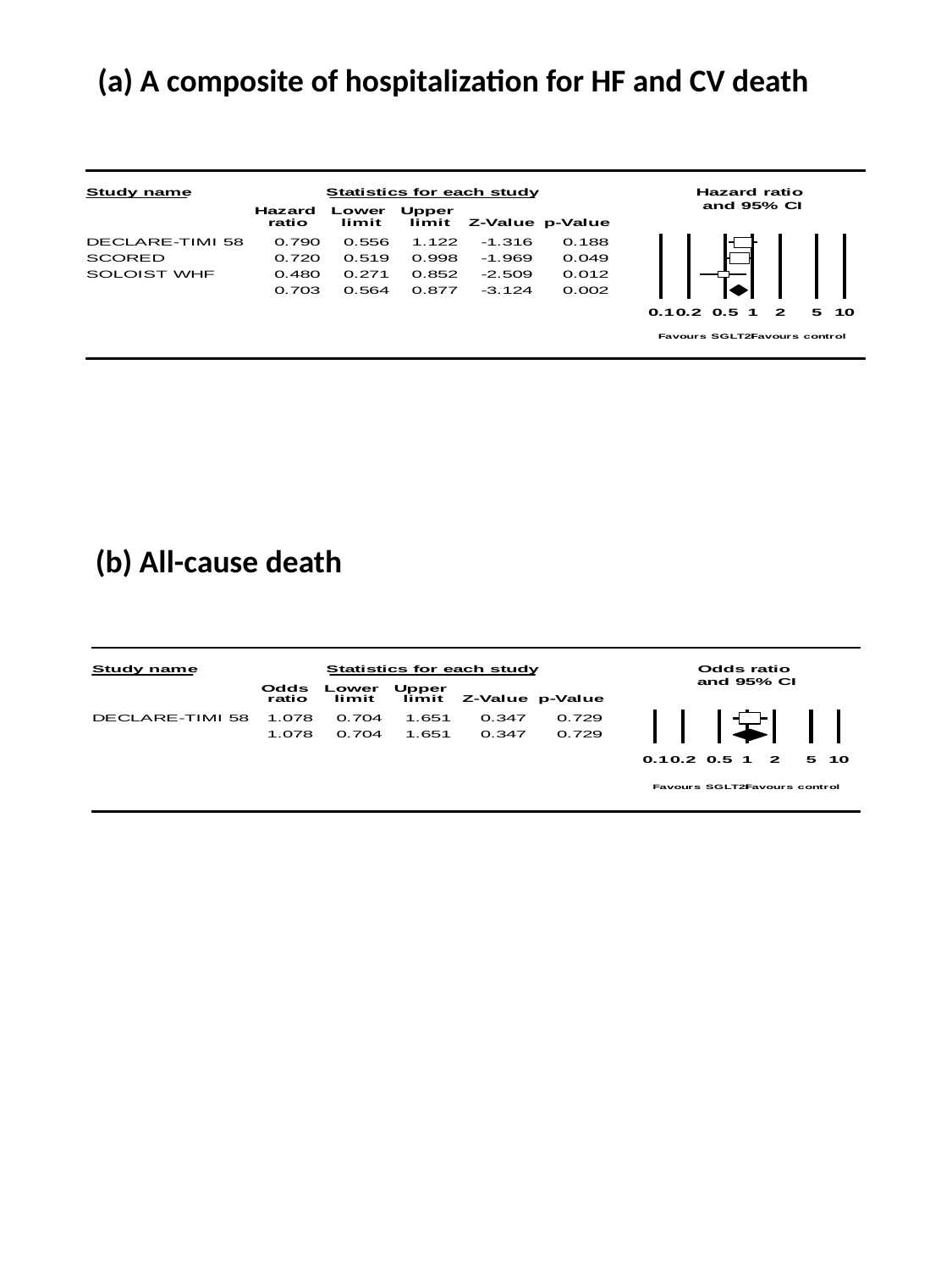

(a) A composite of hospitalization for HF and CV death
(b) All-cause death

Supplement: Supplementary Fig. 4 — Forest plots showing the effects of sodium–glucose cotransporter 2 inhibitors (SGLT2i) on a composite of hospitalization for heart failure (HF) and cardiovascular (CV) death (a) and all-cause death (b) for trials that used ejection fraction (EF) ≥50% for the diagnosis of heart failure with preserved EF. [file mmc4.pptx]

## Slide 1
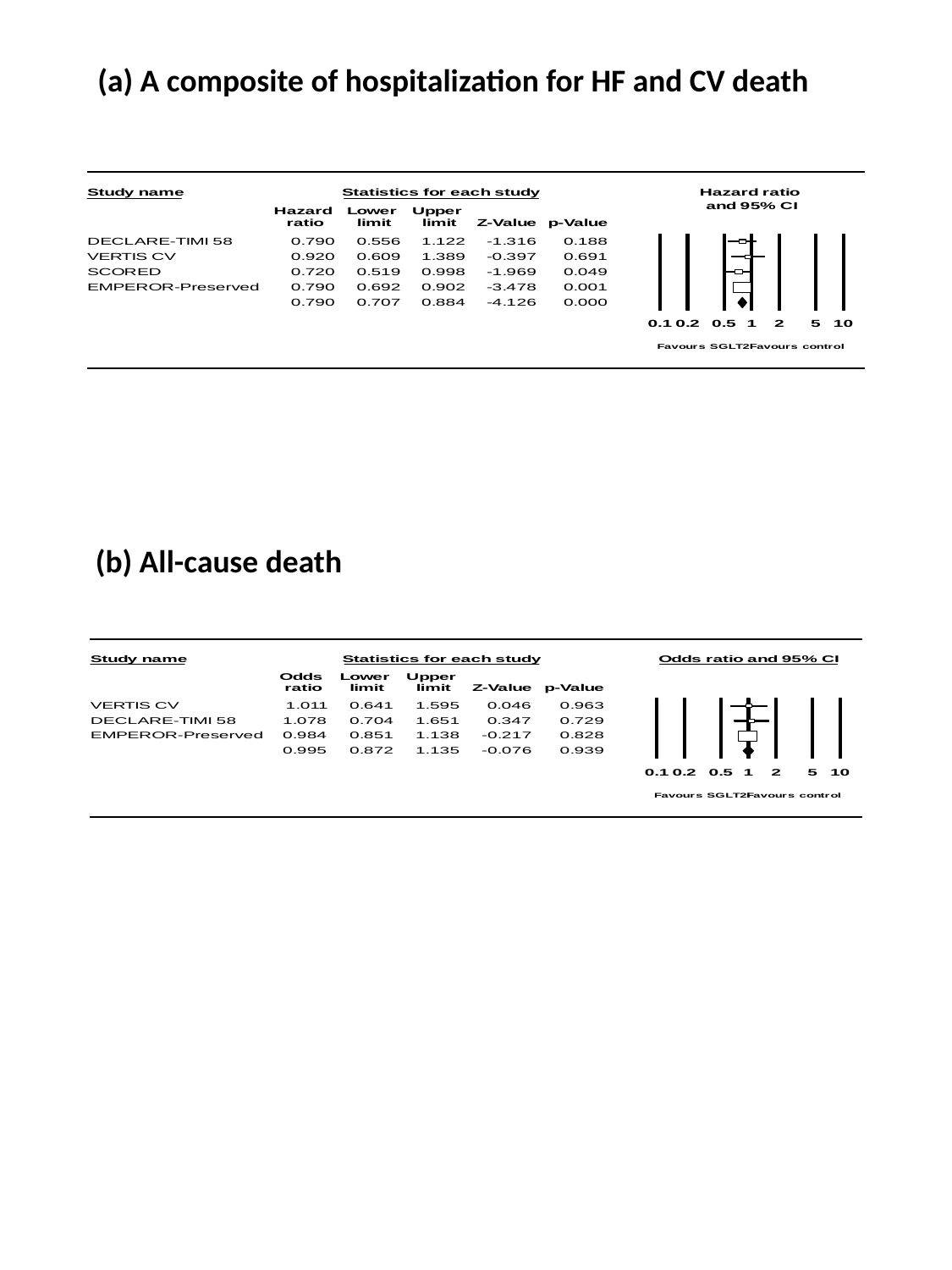

(a) A composite of hospitalization for HF and CV death
(b) All-cause death

Supplement: Supplementary Fig. 5 — Forest plots showing the effects of sodium–glucose cotransporter 2 inhibitors (SGLT2i) on a composite of hospitalization for heart failure (HF) and cardiovascular (CV) death (a) and all-cause death (b) for trials with longer (>1 year) follow-up. [file mmc5.pptx]

## Slide 1
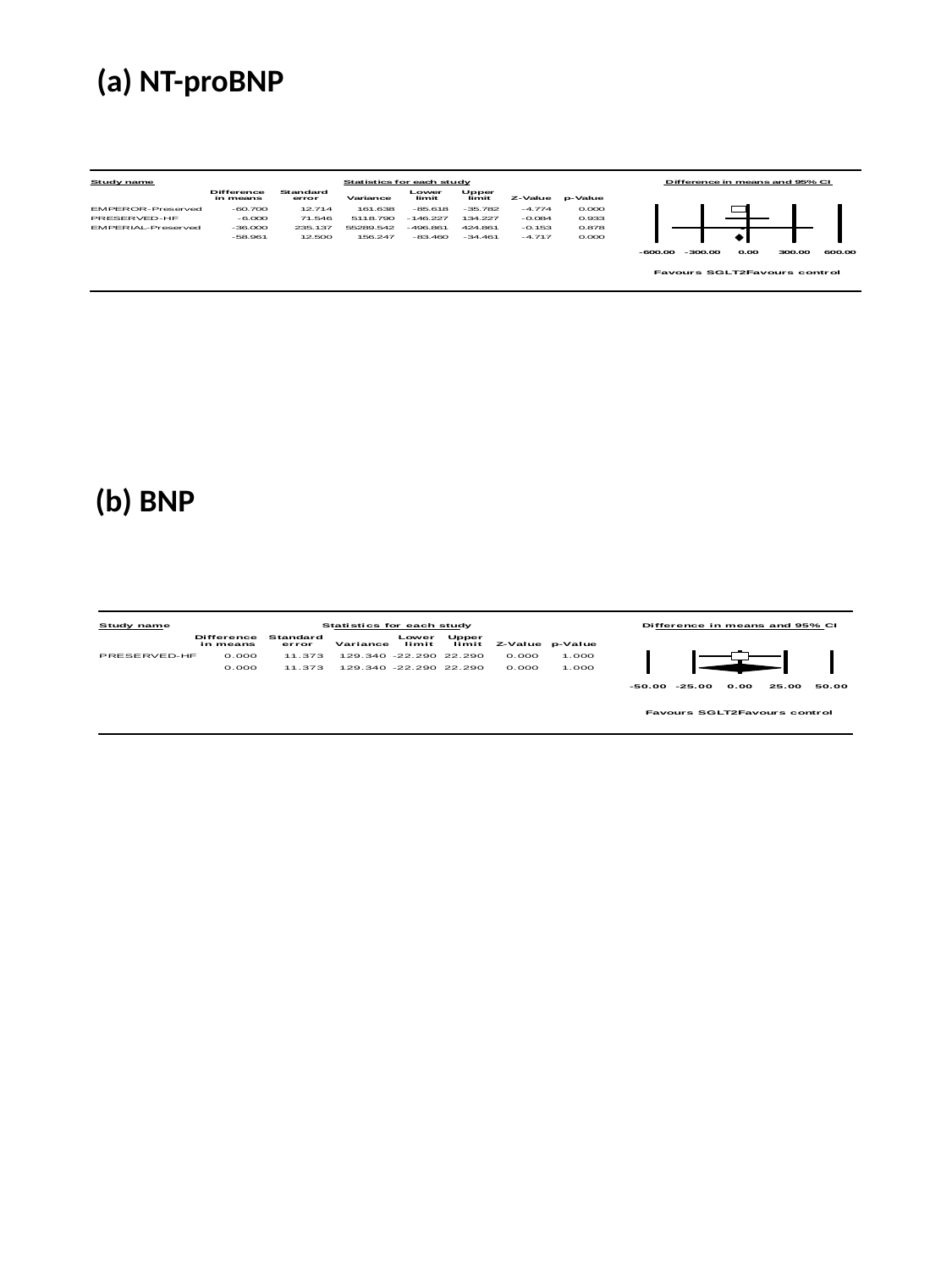

(a) NT-proBNP
(b) BNP

Supplement: Supplementary Fig. 6 — Forest plots showing the effects of sodium–glucose cotransporter 2 inhibitors (SGLT2i) on N-terminal pro-B-type natriuretic peptide levels (NT-proBNP; pg/ml; a), plasma B-type natriuretic peptide levels (BNP; pg/ml; efb) for trials that used placebo as control. [file mmc6.pptx]
