# Supplementary material for: Sodium-glucose cotransporter 2 inhibitors in heart failure with preserved ejection fraction: A meta-analysis of randomized controlled trials
Source: Int J Cardiol Heart Vasc. 2022 Aug 11;42:101103. doi: 10.1016/j.ijcha.2022.101103 (PMC9399288; doi:10.1016/j.ijcha.2022.101103)
Supplement: Supplementary Table 1 [file mmc7.docx]

**Supplementary Table 1. Patient characteristics**

| Study | Mean  age,  year | Women | NYHA  class >II | Mean  BMI,  Kg/m^2^ | Mean  EF, % | Diabetics | CAD | AF | HTN | BB | ACE-I  or ARB | MRA | ARNI | Loop  diuretics |
| --- | --- | --- | --- | --- | --- | --- | --- | --- | --- | --- | --- | --- | --- | --- |
| DECLARE-TIMI 58^*^ | 65^†^ | 43% | 6% | 33^†^ | 55^†^ | 100% | 87% | NR | 96% | 77% | 85% | 14% | NR | 35% |
| VERTIS CV | 64 | 36% | 5% | 33 | NR | 100% | 88% | NR | 95% | 78% | 85% | 11% | NR | 23% |
| SCORED | NR | NR | NR | NR | NR | 100% | NR | NR | NR | NR | NR | NR | NR | NR |
| SOLOIST WHF | NR | NR | NR | NR | NR | 100% | NR | NR | NR | NR | NR | NR | NR | NR |
| CANDLE | NR | NR | NR | NR | NR | 100% | NR | NR | NR | NR | NR | NR | NR | NR |
| MUSCAT-HF | 63 | 38% | 2% | 25.4 | 58 | 100% | 59%^¶^ | 20% | 82% | 61% | 59% | 24% | 0% | 23% |
| CANONICAL | 76 | 33% | 6% | 25.0 | 62 | 100% | NR | 34% | 90% | 71% | 23%  or 58% | 17% | 0% | 44% |
| EMPEROR-  Preserved | 72 | 45% | 18% | 29.8 | 54 | 49% | 35% | 51% | 90% | 86% | 81% | 37% | 2% | NR |
| PRESERVED-  HF | 70 | 57% | 42% | 35^†^ | 60 | 56% | 19% | 53% | NR | 73% | 61% | 36% | 2% | 88% |
| EMPERIAL-  Preserved | 74^†^ | 43% | 23% | 29.6 | 53^†^ | 51% | 41% | 30% | NR | 89% | 75% | 33% | 3% | 72% |
| EXCEED | 71 | 40% | 0% | NR | 61 | 100% | NR | 0% | NR | NR | NR | NR | NR | NR |

ACE-I indicates angiotensin converting enzyme inhibitor; AF, atrial fibrillation; ARB, angiotensin receptor blocker; ARNI, angiotensin-receptor neprilysin inhibitor; BB, beta-blocker; BMI, body mass index; CAD, coronary artery disease; EF, ejection fraction; HTN, hypertension; MRA, mineral corticoid receptor blocker; NR, not reported; NYHA, New York Heart Association.

^*^reported as HF without known reduced EF.

†reported as median.

^¶^reported as the prevalence of cardiovascular diseases.
